# Effect of magnesium supplementation on emergence delirium and postoperative pain in children undergoing strabismus surgery: a prospective randomised controlled study

**DOI:** 10.1186/s12871-020-01192-7

**Published:** 2020-11-18

**Authors:** Ji-Hyun Lee, Seungeun Choi, Minkyoo Lee, Young-Eun Jang, Eun-Hee Kim, Jin-Tae Kim, Hee-Soo Kim

**Affiliations:** Department of Anaesthesiology and Pain Medicine, Seoul National University Hospital, Seoul National University College of Medicine, # 101 Daehakno, Jongnogu, Seoul, 03080 Republic of Korea

**Keywords:** Emergence delirium, Magnesium, Ophthalmologic surgical procedure, Paediatrics, Pain

## Abstract

**Background:**

The benefits of intraoperative magnesium supplementation have been reported. In this prospective, randomized study, the effects of magnesium supplementation during general anaesthesia on emergence delirium and postoperative pain in children were evaluated.

**Methods:**

A total of 66 children aged 2 to 5 years who underwent strabismus surgery were assigned to the magnesium or to the control group. Preoperative anxiety was assessed using the modified Yale Preoperative Anxiety Scale. After anaesthesia induction, the magnesium group received an initial loading dose of 30 mg/kg magnesium sulphate over 10 min and, then, continuous infusion of 10 mg/kg per h until 10 min before the end of the surgery. The control group received an equal volume of normal saline via the same regimen. The Paediatric Anaesthesia Emergence Delirium (PAED) score, pain score, and respiratory events were assessed at the postanaesthetic care unit.

**Results:**

Data obtained from 65 children were analyzed. The PAED and pain scores of the two groups did not differ significantly. There were 26 of 33 (78.8%) and 27 of 32 (84.4%) children with emergence delirium in the control and the magnesium groups, respectively (odds ratio 0.69, 95% CI 0.19–2.44; *p* = 0.561). The preoperative anxiety score was not significantly correlated with the PAED score. The incidence of respiratory events during the emergence period did not differ significantly between the two groups.

**Conclusions:**

Magnesium supplementation during anaesthesia had no significant effects on the incidence of emergence delirium or postoperative pain in children undergoing strabismus surgery.

**Trial registration:**

ClinicalTrials.gov (NCT03132701). Prospectively registered May 8, 2017.

## Background

Emergence delirium after general anaesthesia is a common phenomenon, and rates > 80% have been reported in children [1]. It has been associated with fast-acting inhalation anaesthetics, such as sevoflurane or desflurane, male sex, ophthalmology and otolaryngology procedures, younger age, and preoperative anxiety, and its incidence has been shown to be reduced by intraoperative opioids, benzodiazepine, and alpha 2 adrenergic agonists [2].

Magnesium is the fourth most common cation in the human body and known to be a modulator of transmembrane ion transport and energy metabolism [3]. Magnesium sulphate is an N-methyl-D-aspartate receptor antagonist that is used to treat hypomagnesemia, preeclampsia and polymorphic ventricular arrhythmia, and also used as an anti-convulsive agent. Additionally, the use of magnesium during the perioperative period has been associated with increased sedation, analgesia, reduced administration of neuromuscular blockade agents, and the prevention of ischemic-reperfusion injury [4, 5]. In children, intraoperative infusion of magnesium may reduce emergence delirium after adenotonsillectomy [6] and hernia repair [7]. However, Apan et al. [8] reported that magnesium supplementation had no influence on the incidence of emergence delirium in paediatric patients.

Perioperative hypomagnesemia is common because some intravenous fluid solutions administered during fasting, including Hartman solution and normal saline, do not contain magnesium [9]. Therefore, magnesium supplementation during anaesthesia can reduce the required amounts of sedatives, analgesics, or neuromuscular blocking agents, and contribute to improved postoperative outcomes [10]. We hypothesised that magnesium supplementation in paediatric patients may also be associated with reductions in the amounts of anaesthetics and analgesics required, and reduced postoperative emergence delirium. Our aim was to evaluate the effects of magnesium supplementation during general anaesthesia on emergence delirium and postoperative pain in children undergoing strabismus surgery. Other post-anaesthesia recovery parameters, including nausea, vomiting, and respiratory complications, were also assessed.

## Methods

### Study population

This single-centre study was performed at the Seoul National University Children’s Hospital, a tertiary children’s hospital in South Korea. Sixty-six children aged 2–5 years (American Society of Anesthesiologists physical status I or II) who were scheduled for elective strabismus surgery under general anaesthesia were included. The exclusion criteria were as follows: history of hypersensitivity and malignant hyperthermia, currently taking an anti-epileptic drug, known myasthenia gravis, myasthenic syndrome, neuromuscular disease, arrhythmia, moderate cardiovascular, pulmonary, hepatobiliary, or renal disease, or overweight (body mass index > 85 percentile). The study protocol was approved by the Institutional Review Board of the Seoul National University Hospital (approval number: H1703–110-840; date of approval: May 8, 2017) and was registered at https://clinicaltrials.gov (number: NCT03132701; principal investigator: Hee-Soo Kim; date of registration: April 9, 2017). The anaesthesiologists involved in the study obtained written informed consent from the parents or their guardians after explaining the study protocol to them.

### Group allocation

This study was a randomised, controlled, parallel-designed trial. Following a simple randomisation procedure (computerised random number; https://www.randomizer.org), the children were allocated to the magnesium or the control group. An anaesthetic nurse who was not involved in the study prepared coded and sealed, opaque envelopes, and the allocation ratio was 1:1. Immediately before induction of anaesthesia, she prepared the study drug, either magnesium or normal saline, according to group allocation. The patients, attending anaesthesiologists, and two researchers (LJH and CSE) who assessed the preoperative anxiety and outcomes including delirium scale and pain score were blinded to group allocations.

### Anaesthesia and study protocol

All strabismus surgeries were performed as day surgeries, and started before 11 am according to the day-surgery policy of our centre. All patients had the following minimum fasting time; 8 h for heavy meal, 6 h for light meal and non-human milk, and 2 h for clear fluid. An intravenous line was established in all children before anaesthetic induction, and Ringer’s lactate solution was administered before and during anaesthesia.

The extent of preoperative anxiety was assessed using the modified Yale Preoperative Anxiety Scale (m-YPAS) [11] when patients and their parents arrived at the reception area of the operating room. Anaesthesia induction was commenced with atropine 0.02 mg/kg, propofol 2.5 mg/kg after electrocardiography monitoring, pulse oximetry, and non-invasive blood pressure determination. No other systemic or local analgesics, such as opioids or eye drops, were used during the induction period. Facemask ventilation was performed with sevoflurane and 100% oxygen and, then, a flexible laryngeal mask airway (Marshall flexible LAD®, Marshall Airway Products Ltd., Radstock, UK) was inserted. The intracuff pressure of the laryngeal mask airway was adjusted within 30–40 cmH_2_O using a cuff manometer (VBM Medizintechnik GmbH, Sulz am Neckar, Germany). Neuromuscular blocking agents were not used basically, but allowed as needed for the maintenance of anaesthesia. Mechanical ventilation was commenced using volume-controlled mode with tidal volume of 8 ml/kg without positive end-expiratory pressure. During anaesthesia, sevoflurane concentration was controlled to maintain a bispectral index target between 40 and 60.

At the beginning of anaesthesia induction, the children in the magnesium group received an initial intravenous loading dose of 30 mg/kg magnesium sulphate over 10 min (0.3 ml/kg), then continuous infusion of 10 mg/kg (0.1 ml/kg) per h until 10 min before the end of surgery. The control group received an equal volume of normal saline via the same infusion regimen. Preparations of ephedrine and atropine were readied for possible complications such as hypotension and bradycardia.

At the end of surgery, propacetamol 30 mg/kg was administered to all patients. After gentle pharyngeal suction, the laryngeal mask airway was removed and the patient was transferred to the postanaesthetic care unit (PACU). Complications during the emergence period, such as laryngospasm, bronchospasm, desaturation, breath holding, and coughing were recorded.

**Fig. 1:**
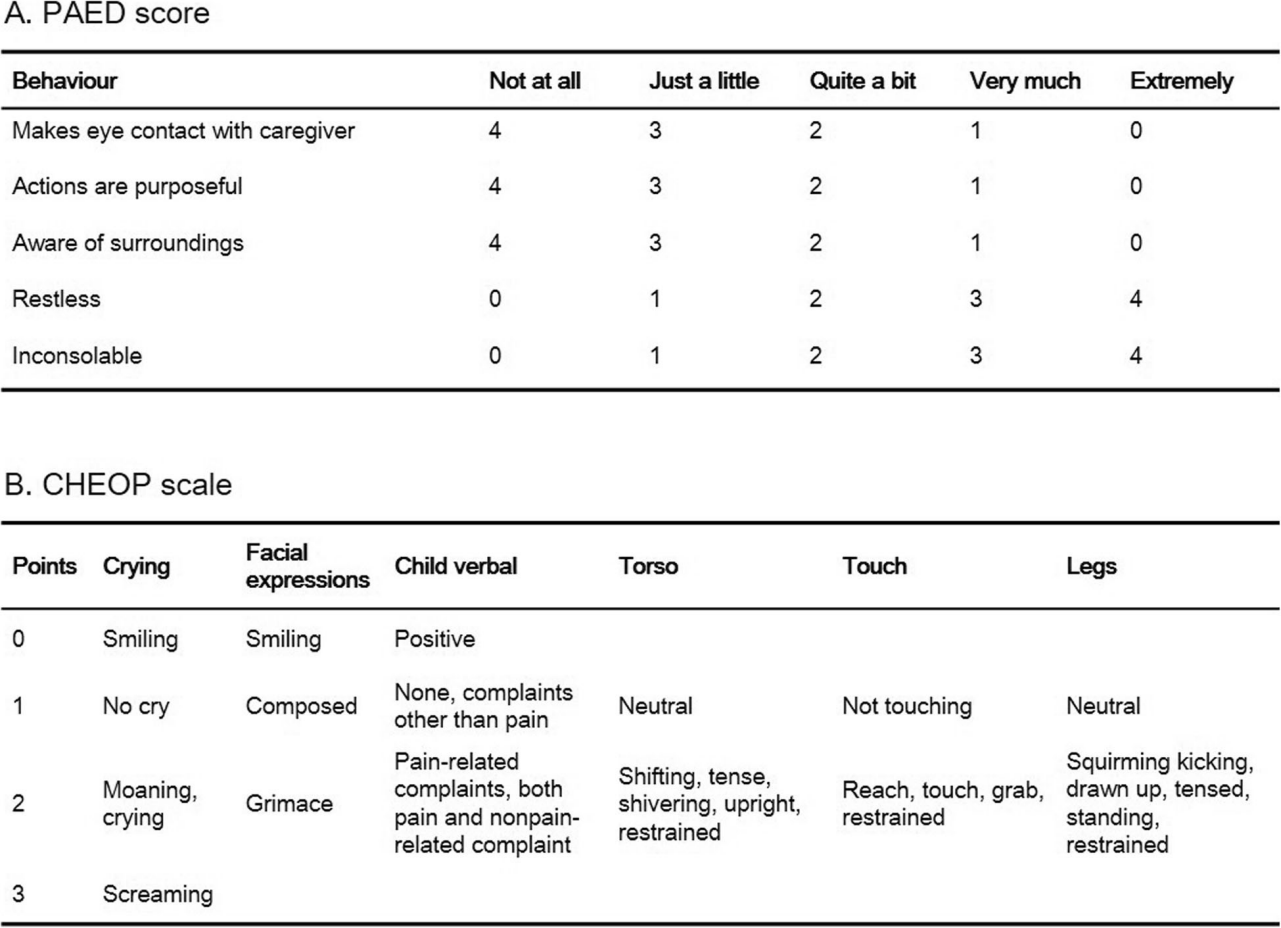
Pediatric Anaesthesia Emergence Delirium score (**a**) and Children’s Hospital of Eastern Ontario Pain scale (**b**)

At the PACU, all patients’ vital signs, including heart rate, noninvasive blood pressure, respiratory rate, and peripheral oxygen saturation, were continuously monitored and recorded every 5 min. The Paediatric Anaesthesia Emergence Delirium (PAED) score (Fig. 1a) [12] and other complications were assessed on arrival in the PACU and every 10 min until discharge from the PACU. The pain score (Children’s Hospital of Eastern Ontario Pain scale; CHEOPS, Fig. 1b [13]) was also assessed on arrival in the PACU, at 30 min after arrival, and at discharge. When the PAED score was greater than 12, which was considered as the presence of emergence derlirium [14], nalbuphine 0.1 mg/kg was administered intravenously. When the CHEOPS score was more than 7, ketorolac 0.5 mg/kg was administered intravenously if the patients did not receive nalbuphine. Patients were discharged from the PACU when they had a modified Aldrete score greater than 9. The patients were continuously monitored for complications, including nausea, vomiting and respiratory concerns. Symptoms of hypermagnesaemia, such as hypotension, bradycardia, lethargy, paralysis and headache, were also monitored until the patients were discharged from the ambulatory surgery centre.

### Statistical analysis

The primary outcome of this study was the PAED score in both groups. The occurrence of emergence delirium was defined when the PAED scores were ≥ 12 at any time point in the PACU. The secondary outcomes included the incidence of emergence delirium during PACU stay, CHEOPS score, incidence of nausea, vomiting and respiratory complications, and length of PACU stay.

The sample size was calculated based on a previous study [6] that investigated the effects of intra-operative magnesium sulphate administration on the incidence of emergence delirium in children who had undergone adenotonsillectomy. In that study, the respective rates of emergence delirium in the magnesium and the control group were 36 and 72%, respectively. Thus, the sample size required for our study was calculated to be approximately 30 patients per group, with an alpha error of 0.05 and a power of 0.8, as determined via PASS software 2008 (version 8.0.16; NCSS statistical software, Kaysville, UT, USA). Based on an attrition rate of up to 10%, a total of 66 patients were enrolled.

All data were analysed using SPSS for Windows (version 23.0; IBM Corp., Armonk, NY, USA). Data normality was assessed using the Kolmogorov–Smirnov test. Categorical variables are expressed as numbers and percentages, and continuous variables as means and standard deviations or medians and interquartile ranges. The Chi-square test was used to assess the significance of categorical data comparisons, and the Fisher’s exact test was used when the expected count of > 20% cells was less than five. The Pearson’s correlational analysis was performed to assess the correlation between the preoperative anxiety and PAED scores. The Student’s *t*-test or the Mann–Whitney rank-sum test were used to examine the significance of continuous data comparisons. Repeated measures data were analysed by the analysis of variance, and the Bonferroni’s correction was used for post-hoc analysis. All *p* values < 0.05 were considered statistically significant.

## Results

**Fig. 2:**
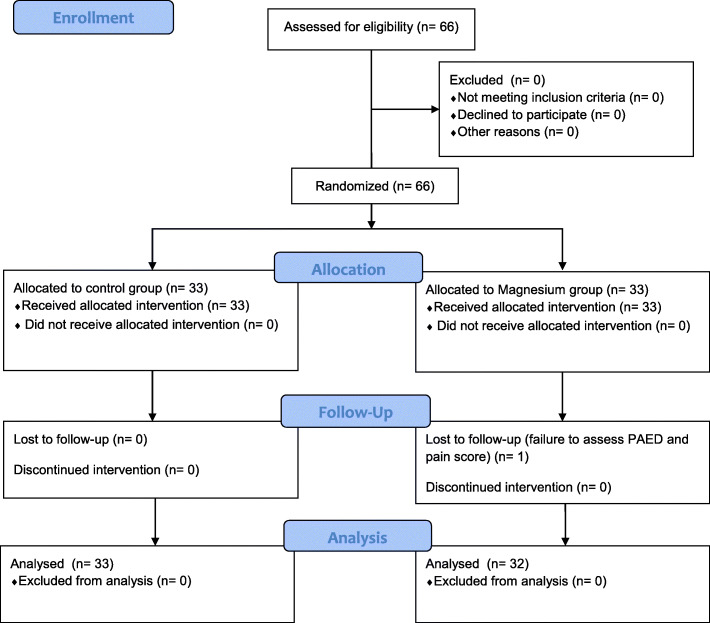
CONSORT diagram

A total of 66 paediatric patients were initially enrolled from June to December 2017, and randomised into two groups. One patient in the magnesium group was subsequently excluded due to a lack of PAED and pain score assessment. Therefore, data from 65 children (33 and 32 in the control and the magnesium group, respectively) were analysed (Fig. 2).

Table 1 shows the demographic data of patients in the magnesium and the control groups. There were no significant differences in baseline characteristics including the preoperative m-YPAS scores between the two groups.

**Table 1:** Demographic characteristics of the study population

	Control (***n*** = 33)	Magnesium (***n*** = 32)	***P*** value
**Age (years)**	4.4 ± 0.9	4.0 ± 1.2	0.218
**Sex (M/F, %)**	14/19 (42.4/57.6)	14/18 (43.8/56.3)	0.914
**Height (cm)**	108.6 ± 8.0	105.4 ± 9.0	0.135
**Weight (kg)**	18.4 ± 3.0	17.4 ± 3.1	0.176
**Operation time (min)**	25 (20–35)	20 (15–28.75)	0.138
**Anesthesia time (min)**	44.8 ± 2.6	40.8 ± 10.9	0.180
**Size of laryngeal mask airway (2/2.5, %)**	26/7 (78.8/21.2)	28/4 (87.5/12.5)	0.511
**m-YPAS**			
Activity	2.0 (1.0–2.0)	2.0 (1.0–2.0)	0.281
Vocalization	2.0 (1.0–3.0)	2.0 (1.0–3.0)	0.781
Emotional expressivity	2.0 (1.25–3.0)	2.0 (1.0–3.0)	0.300
State of apparent arousal	2.0 (1.0–2.75)	1.0 (1.0–2.0)	0.534
Use of parents	2.0 (1.25–3.0)	2.0 (1.0–3.0)	0.501
Total score	47.5 (30.4–58.0)	41.7 (28.3–60.0)	0.465

Data are presented as mean ± standard deviations, median (interquartile ranges) or number (percentage)

*m-YPAS* Modified Yale Preoperative Anxiety Scale

**Fig. 3:**
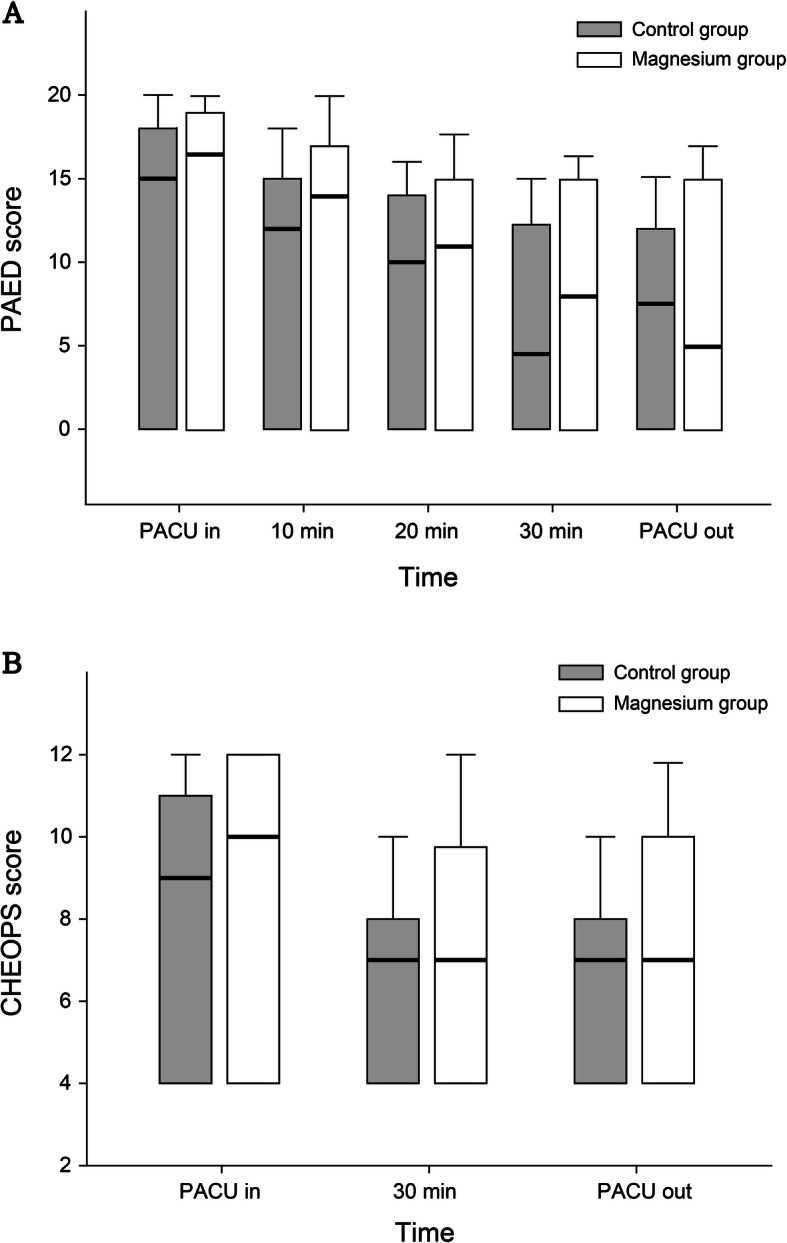
The PAED score (**a**) and the CHEOPS score (**b**) over time in both groups. The boundary of the box indicates the 25th and 75th percentile, and a bold line within the box marks the median. The error bars indicate the 10th and 90th percentiles. PAED, Pediatric Anaesthesia Emergence Delirium; CHEOPS, Children’s Hospital of Eastern Ontario Pain Scale

Table 2 shows the postoperative PAED and the CHEOPS scores in both groups. The median PAED scores over time did not differ significantly (*p* = 0.806) between the two groups. Figure 3a shows the PAED scores over time in both groups. The incidences of emergence delirium were 26 (78.8%) and 27 (84.4%) in the control and the magnesium groups, respectively (OR 0.69, 95% CI 0.19–2.44, *p* = 0.56).

**Table 2:** Postoperative PAED and CHEOPS scores in both groups

	Control (***n*** = 33)	Magnesium (***n*** = 32)	***P*** value
**PAED score**			0.806*
PACU in	15.0 (0–18.0)	16.5 (0–19.0)	0.417
10 min	12.0 (0–15.0)	14.0 (0–17.0)	0.313
20 min	10.0 (0–14.0)	11.0 (0–15.0)	0.253
30 min	4.5 (0–12.25)	8.0 (0–15.0)	0.171
PACU out	7.5 (0–12.0)	5 (0–15.0)	0.967
**CHEOPS score**			0.623*
PACU in	9.0 (4.0–11.0)	10.0 (4.0–12.0)	0.390
30 min	7.0 (4.0–8.0)	7.0 (4.0–9.75)	0.199
PACU out	7.0 (4.0–8.0)	7.0 (4.0–10.0)	0.664

Data are presented as median (interquartile ranges)

*P value from repeated measures ANOVA

*CHEOPS* Children’s Hospital of Eastern Ontario Pain scale, *PACU* Postanaesthetic are unit, *PAED* Pediatric anesthesia emergence delirium

The CHEOPS scores over time did not differ significantly between the two groups (Fig. 3b). No rescue analgesics were administered in the PACU in either group. The m-YPAS score was not significantly correlated with the PAED score at any time-point (PACU entry, *r* = 0.1, *p* = 0.438; after 10 min, *r* = 0.12, *p* = 0.336; after 20 min, *r* = 0.04, *p* = 0.750; after 30 min, *r* = 0.07, *p* = 0.599; exiting PACU, *r* = 0.13, *p* = 0.343).

Table 3 shows the intraoperative variable data in both groups. The peak inspiratory pressure, the mean sevoflurane concentration, and mean bispectral index value during surgery did not differ significantly in the two groups. There were no differences in the intraoperative mean heart rate and blood pressures between the two groups. During emergence, the diastolic and mean blood pressures were higher in the control group than in the magnesium group (diastolic blood pressure: 68 [15] vs 60 [11] mmHg, mean differences [95% CI], 8 [3–13] mmHg, *p* = 0.004; mean blood pressure: 84 [13] vs 76 [10] mmHg, mean differences [95% CI], 7 [1–13] mmHg, *p* = 0.015).

**Table 3:** Intraoperative and postoperative variables of both groups

	Control (***n*** = 33)	Magnesium (***n*** = 32)	***P*** value
**Number of attempts for laryngeal mask airway insertion**	1 (1–1)	1 (1–1)	0.965
**Number of cases for laryngeal mask repositioning**	0	0	.
**Mean sevoflurane concentration (vol%)**	2.7 ± 0.3	2.5 ± 0.4	0.707
**Intraoperative mean BIS value**	50 ± 4	49 ± 3	0.192
**Peak inspiratory pressure (cmH**_**2**_**O)**			
Maximum pressure	15.2 ± 4.6	15.6 ± 4.8	0.790
Minimum pressure	12.7 ± 2.2	13.3 ± 2.4	0.359
**Intraoperative hemodynamic parameters**			
Heart rate (bpm)	129 ± 18	134 ± 13	0.219
Systolic blood pressure (mmHg)	92 ± 7	93 ± 10	0.611
Diastolic blood pressure (mmHg)	49 ± 8	48 ± 8	0.391
Mean blood pressure (mmHg)	66 ± 9	63 ± 7	0.231
**Hemodynamic parameters during emergence**			
Heart rate (bpm)	122 ± 19	124 ± 14	0.628
Systolic blood pressure (mmHg)	107 ± 15	101 ± 11	0.087
Diastolic blood pressure (mmHg)	68 ± 15	60 ± 11	0.004*
Mean blood pressure (mmHg)	84 ± 13	76 ± 10	0.015*
**Time from surgery end to PACU admission (min)**	5.9 ± 2.2	6.1 ± 2.8	0.733
**Respiratory event during emergence**	8 (24.2%)	8 (25.0%)	1.0
Laryngospasm	0	1 (3.1%)	1.0
Desaturation	4 (12.1%)	2 (6.3%)	0.672
Breath holding	2 (6.1%)	0	0.492
Coughing	4 (12.1%)	5 (15.6%)	1.0
**Length of PACU stay**	34.2 (3.1)	34.5 (5.4)	0.771

Data are presented as median (interquartile ranges), mean ± standard deviations or number (percentages)

*P < 0.05 between the control and magnesium groups

*BIS* Bispectral index, *PACU* Postanaesthetic care unit

In the PACU, no patient experienced nausea and vomiting. There were no significant complications during the PACU stay in both groups. Moreover, the length of stay at the PACU was similar between the two groups.

## Discussion

In this study, we found that magnesium supplementation during strabismus surgery had no significant effect on the incidence of emergence delirium and postoperative pain in children. In addition, there was no significant difference in respiratory complications, length of PACU stay, and other intraoperative parameters between the magnesium and control groups. Lastly, there were no complications associated with intraoperative magnesium supplementation.

Although the mechanism of emergence delirium after general anaesthesia has not been clearly defined, there are some well-known risk factors including young age, no previous surgery, ophthalmology procedures, otorhinolaryngology procedures, volatile anaesthetics such as sevoflurane, and preoperative anxiety [2, 15]. In addition, postoperative pain evidently may have a role in emergence delirium because the administration of analgesics, including opioids, has been reported to prevent the emergence delirium in children [16, 17].

In this report, the term ‘emergence delirium’ was used to describe the behavioural change following general anaesthesia to maintain consistency with the referenced reports. However, there have been inconsistent use of the terms ‘delirium’ and ‘agitation’ in the literature. Emergence delirium refers to an altered state of consciousness, which begins with emergence from anaesthesia and continues through the early recovery period. On the other hand, emergence agitation is an umbrella term, and is affected by emergence delirium, pain, and several other factors [12, 18]. In this study, PAED scores were used to assess ‘delirium’ apart from pain.

The activation of N-methyl-D-aspartate (NMDA) receptor changes the excitatory properties of neurons that can induce seizures, and as magnesium is an NMDA receptor antagonist it can have sedative and anti-convulsive effects. In addition, magnesium has analgesic effects and can lead to a reduction in perioperative opioid consumption by blocking the NMDA receptors, which are involved in nociception [9]. Therefore, considering the effect of magnesium and the mechanism of emergence delirium, it is reasonable to expect that magnesium may reduce emergence delirium.

There are limited data pertaining to the association between magnesium supplementation and reduced emergence delirium [6, 7]. According to Abdulatif et al. [6], 30 mg/kg bolus intravenous magnesium sulphate followed by 10 mg/kg per h during sevoflurane anaesthesia reduced the incidence of emergence delirium with a relative risk of 0.51 in children undergoing adenotonsillectomy [6]. Bondok et al. [7] reported that no emergence delirium occurred in male children who received magnesium supplementation undergoing elective inguinal herniorrhaphy.

To the best of our knowledge, this is the first study that evaluated the effect of magnesium supplementation in children undergoing ophthalmic surgery. There were some differences between the present study and previous studies. In two studies demonstrating the beneficial effect of magnesium, combination analgesic therapy was used with opioids, non-steroidal anti-inflammatory drugs, and regional block [6, 7]. In our study, we used propacetamol only for pain control to minimise the confounding effects of analgesics. The differences in the analgesic use and type of surgery might contribute to the higher incidence of emergence delirium in this study (approximately 80%) compared to that in previous studies (35% [6] and 50% [7]).

There are several possible reasons for the non-significant association between magnesium and emergence delirium observed in this study. Magnesium concentrations may have been within the normal range even in the control group, as it was reported by Apan et al. [8] Therefore, the additional increase in magnesium concentration may not have functioned to reduce emergence delirium or pain. In addition, genetic factors may also be relevant. Genetic differences in pain sensitivity [19], responses to analgesics due to alterations of pharmacokinetic and pharmacodynamic parameters [20, 21], and emergence delirium [22] have been reported. Additionally, there may be differences associated with race. Finally, there might be other factors that influenced the occurrence of emergence delirium. According to Joo et al., emergence delirium was associated with the level of invasiveness of the procedure in children undergoing ophthalmic surgery [23]. There were wide variations in operating time in this study, and we speculated that complexity of surgery, surgical skill, or operation time might be potential factors affecting the recovery characteristics.

Preoperative anxiety can affect emergence delirium [2, 24], and several studies have reported their association [25, 26]. However, in this study, we could not find a correlation between the m-YPAS and PAED scale. Our result was similar to that of a previous study, suggesting that visual disturbances might play a greater role in emergence delirium compared with preoperative anxiety [23].

Previous studies concluded that perioperative adjuvant magnesium sulphate administration reduced the requirements for nondepolarizing neuromuscular blockers [27–29]. We also expected that intraoperative magnesium supplementation could reduce the peak inspiratory pressure and spontaneous respiratory effort, as magnesium has property for potentiation of muscle relaxation and, thus, no neuromuscular blockade was used in the present study [10]. However, we could not find the group difference in the peak inspiratory pressure and incidence of spontaneous respiratory effort.

On the other hand, the control group showed higher diastolic and mean blood pressure during the emergence period when compared to the magnesium group. Magnesium has vasodilatory effects, and is known to reduce the need for alpha-beta blockers [9]. Hypotension is one of the complications of magnesium administration, which can occur when the serum magnesium level exceeds 3–4 mg/dl [9]. Although we could not assess the serum magnesium level, there were no patients with significant hypotension. Sympathetic tone usually increases during the emergence period, and we speculated that magnesium may prevent the further increase in blood pressure in the magnesium group.

Our study had some limitations. The sample size was too small with regard to the statistical power, as it was calculated based on previous studies, in which there were significant differences between the control and magnesium groups [6]. Additionally, the serum magnesium levels were not evaluated before and after the administration of magnesium sulphate in all patients. Magnesium supplementation can be helpful when hypomagnesemia is obvious, but hypomagnesemia may not be commonly associated with short operations and minimal fasting times [8]. Second, there might be a possibility of hypermagnesemia and safety issue should be considered. The possible adverse effects of hypermagnesemia are bradycardia and hypotension. However, there were no cases of clinical consequences and no need for treatment withdrawal in paediatric population [9]. In addition, there were no critical incidents related to magnesium supplementation in the present study. Third, the incidence of emergence delirium was higher than expected when calculating the sample size. This may be associated with the relatively high pain scores in our patients. When preschool children with emergence delirium have pain, pain-related behaviour could be assessed as emergence delirium [30]. Additionally, postoperative nausea and vomiting may present as agitation. Finally, the PAED and pain scores were assessed only in the PACU. The data would have been more informative and valuable if the patients were followed up for emergence delirium and pain in the first 24 h postoperatively.

In conclusion, in our study magnesium supplementation had no significant effect on emergence agitation or postoperative pain in children who had undergone strabismus surgery. Other strategies to minimise emergence agitation in children should also be investigated.

## Data Availability

The datasets used and/or analyzed during the current study are available from the corresponding author on reasonable request.

## Abbreviations

BIS: Bispectral index
CHEOPS: Children’s Hospital of Eastern Ontario Pain scale
m-YPAS: Modified Yale Preoperative Anxiety Scale
NMDA: N-methyl-D-aspartate
PACU: Postanaesthetic care unit
PAED: Paediatric Anaesthesia Emergence Delirium
